# Novel Surgical Approach for Aphakia and Iridodialysis: Artificial Iris and Scleral-Fixated Lens as a Single Complex

**DOI:** 10.3390/jcm14051599

**Published:** 2025-02-27

**Authors:** Guglielmo Parisi, Agostino Salvatore Vaiano, Claudio Foti, Francesco Gelormini, Federico Ricardi, Fabio Conte, Maria Marenco, Paola Marolo, Enrico Borrelli, Michele Reibaldi

**Affiliations:** 1 Department of Ophthalmology, University of Turin, 10124 Torino, Italy; guglielmoparisi@gmail.com (G.P.); claudio.foti1@gmail.com (C.F.); francesco.gelormini@hotmail.it (F.G.); federico.ricardi@unito.it (F.R.); fabio.conte94@gmail.com (F.C.); paola@marolo.com (P.M.); borrelli.enrico@yahoo.com (E.B.); 2 Department of Ophthalmology, “Santa Croce e Carle” Cuneo’s Hospital, 12100 Cuneo, Italy; vaiano.a@ospedale.cuneo.it (A.S.V.); marencomaria9012@gmail.com (M.M.)

**Keywords:** artificial iris, scleral-fixated IOL, aphakia, iris trauma, partial aniridia, iridodialysis

## Abstract

**Background**: Artificial iris (AI) implantation is an innovative and increasingly utilized surgical procedure for injured eyes with iris trauma. **Methods:** A 76-year-old female and a 34-year-old male presented at the emergency department with a traumatic corneo-scleral laceration and perforated corneal ulcer, respectively. Emergency surgeries were performed to restore ocular integrity. In both cases, a modified surgical technique involving the implantation of an AI was performed; however, two different models of AI were used. **Results**: The AIs were sutured with four stitches directly to the scleral-fixated (SF) intra ocular lens (IOL), and the AI-IOL complexes were implanted, as a single unit, and fixated to the sclera using the lens haptics. Before and after the surgery, patients underwent a comprehensive eye examination, including a visual acuity test. The AI-SF IOL complexes remained well positioned, with no intraocular or extraocular complications observed during the follow-up evaluations of both patients. **Conclusions**: We reported a straightforward and repeatable modified surgical technique for two patients with two models of AI, both sutured to the SF IOL and fixated to the sclera, as a single unit. This approach may serve as an excellent alternative for managing aphakic eyes with extensive iridodialysis or partial aniridia.

## 1. Introduction

Ocular trauma may result from a variety of causes, including sports injuries, motor vehicle accidents, workplace incidents, and routine daily activities. Depending on the type and magnitude of force applied, these injuries may result in scleral wall laceration, corneal wounds, iridial trauma with partial or total loss of the iridial tissue, lens displacement, and potential retinal damage [1,2]. The primary surgical procedure is aimed at preserving the anatomic integrity of the eye. If further enhancements in visual acuity and cosmetic appearance are achievable, additional elective surgery could be contemplated to improve both function and esthetics, especially in instances of iris trauma. For patients with cosmetic concerns and photophobia due to iris trauma, several minimally invasive options are available, including the use of cosmetic contact lenses and dark-tinted spectacles [3]. For partial iris trauma with tissue loss, typically, within two clock hours, a pupilloplasty can be performed [4]. The use of an artificial iris (AI) is reserved for cases where the previously mentioned solutions are ineffective or unsuitable for the patient, such as in instances of extensive iris damage [5]. The implantation of an AI can be performed using various techniques, either in conjunction with or independently of intraocular lens (IOL) implantation. When capsular support is inadequate for standard IOL implantation, a variety of surgical techniques for AI implantation may be considered [6]. One approach involves suturing the AI to a single-piece IOL and then fixating the resulting AI to the sclera. Alternatively, the scleral-fixated IOL (SF-IOL) and the AI can be independently secured to the sclera with separate sutures [7]. Another option is the Canabrava Double-Flanged Technique, which involves the fixation of an integrated AI-IOL construct to the sclera using the three haptics of the AI [8]. For the first time, we describe a modified surgical technique for the implantation of an SF-IOL with AI as a single complex to treat cases of aphakic eyes with iris trauma.

## 2. Case Presentation

### 2.1. CASE 1#

**Figure 1:**
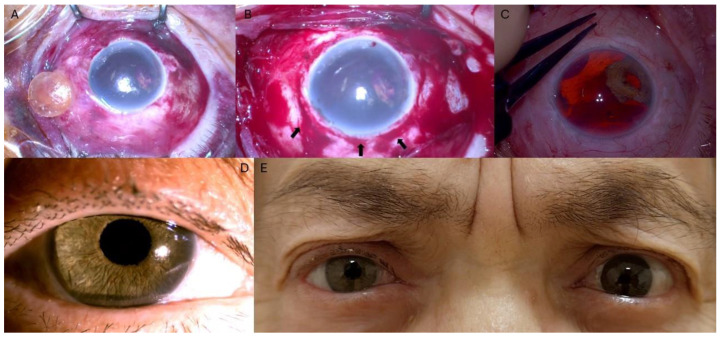
Case #1. (**A**) Biomicroscopic examination revealed an extensive superior scleral laceration, wide iridodialysis, and lens dislocation in right eye. (**B**) 360° peritomy was carried out, revealing a circumferential scleral laceration near the limbus, extending from 11 to 4 o’clock (black arrows). (**C**) Intraoperative image showed aphakia and extensive iridodialysis. (**D**) Seven-month follow-up showed a well-centered AI-SF IOL complex and a clear cornea, demonstrating the esthetic outcome of the procedure (**E**).

A 76-year-old female patient presented with right eye (OD) pain and vision loss following blunt ocular trauma. Biomicroscopic examination revealed an extensive superior scleral laceration, wide iridodialysis, and lens dislocation in the OD. Emergency surgery was performed under general anesthesia. Intraoperatively, the dislocated lens was identified in the nasal quadrant, positioned between the sclera and conjunctiva, and subsequently removed. The scleral laceration was repaired using 7/0 Vicryl sutures (VICRYL^®^, Ethicon LLC, San Lorenzo, PR, USA), successfully restoring ocular integrity (Figure 1A,B). Biomicroscopic examination at the 12-month follow-up (FU) showed a clear cornea, extensive iridodialysis, aphakia, and vitreous hemorrhage (VH), which obstructed a complete retinal assessment. B-scan ultrasonography ruled out retinal detachment and a congested choroid. Twelve months after scleral laceration repair and dislocated lens removal, full anatomical recovery was achieved, and a decision was made to proceed with the combined implantation of an SF-IOL and custom AI (Figure 1C).

#### 2.1.1. Surgical Procedure

**Figure 2:**
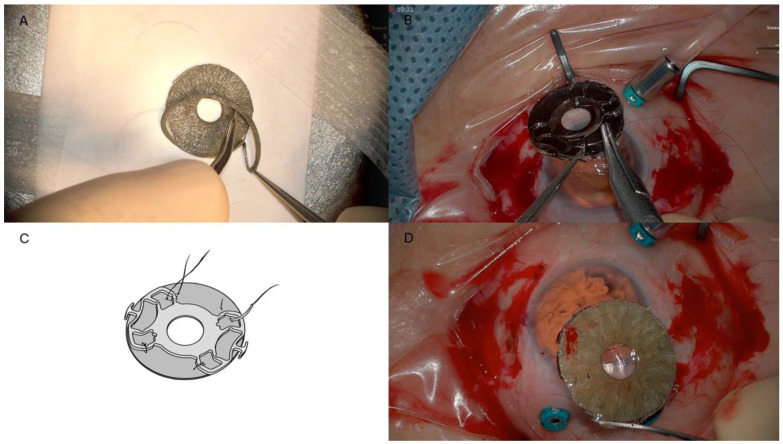
Case #1. (**A**) Preparation of the artificial iris using a trephine, resized to the predetermined diameter based on the white-to-white distance. (**B**) A Prolene suture ran within the iris implant material at the optic–haptic junction and around the all four IOL haptics on both sides. (**C**) Schematic representation of sutures technique in case #1 and #2. The artificial iris is secured to the four haptics of the scleral-fixated IOL using four sutures. (**D**) The AI-SF IOL complex before injection into the anterior chamber.

The assembly of the AI-SF IOL complex was performed as follows: A trephine was used to customize the CUSTOMFLEX^®^ AI implant with fiber (HumanOptics, Erlangen, Germany) based on the patient’s white-to-white (WTW) measurement of 11.80 mm, obtained via optical biometry (IOL Master 500^®^, Carl Zeiss Meditec Inc., Dublin, CA, USA). The AI was then trimmed to a diameter of 11 mm (Figure 2A). Subsequently, the SF-IOL (+20.0 D, Fil SSF^®^, SOLEKO Spa, Rome, Italy, material: hydrophilic acrylic) was secured to the posterior surface of the AI using four 9/0 polypropylene sutures (PRO LENE^®^, Ethicon LLC, San Lorenzo, PR, USA). Each suture ran within the iris implant material at the optic–haptic junction and around the four IOL haptics on both sides (Figure 2B–D). No iridectomy was performed, as a 0.8 mm difference between the AI diameter and the WTW distance was left to ensure adequate aqueous humor flow.

**Figure 3:**
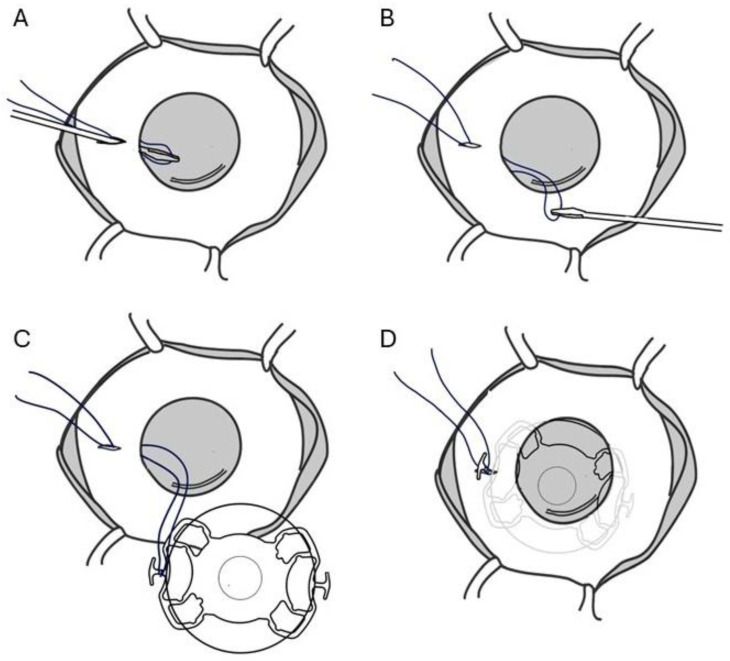
Schematic representation of “Nylon loop technique injection” in case #1. (**A**,**B**) Loop of 10/0 Nylon monofilament suture was inserted through the sclerotomy at 3 o’clock and corneal incision by 23G forceps. (**C**) The monofilament loop was tied to the SF-IOL haptic plug. The partially folded AI-SF IOL complex, with the AI sutured anterior to the SF-IOL, was gently slid through the superior corneal incision into the AC using forceps. (**D**) The SF-IOL haptic was pulled into the scleral pocket using the nylon suture’s loop.

Under peribulbar anesthesia (5 mL of bupivacaine 0.5%, 5 mL lidocaine 2%, and 1500 units of hyaluronidase), three 25-gauge (G) trocars via pars plana were inserted to introduce the infusion BSS cannula and vitrectome (CONSTELLATION^®^ Vision System. Alcon, Geneva, Switzerland). A vitrectomy at 20,000 cuts/min was performed to clear VH and residual iris tissue, following a previous application of diathermy. A conjunctival peritomy was performed at the 3 o’clock and 9 o’clock positions, followed by the creation of two partial-thickness scleral pockets, posteriorly to the limbus, of 2.5 mm with a crescent blade. A 8.0 mm superior limbal incision was made with a 5.5 mm blade. A 23G needle was used to perform opposing sclerectomies, within each scleral pocket, 1.75 mm from the limbus. A loop of 10/0 Nylon monofilament suture (ETHILON^®^, Ethicon LLC, San Lorenzo, PR, USA) was inserted through the sclerotomy at 3 o’clock and corneal incision by 23G forceps (Figure 3A,B). Outside the anterior chamber (AC), the monofilament loop was tied to the SF-IOL haptic plug (Figure 3C). The partially folded AI-SF IOL complex was gently slid through the superior corneal incision into the AC using forceps. The SF-IOL haptic was pulled into the scleral pocket, using the nylon suture’s loop (Figure 3D). The contralateral haptic plug was secured into the opposite scleral pocket by forceps. Five single 7/0 Nylon sutures were placed on the corneal incision. The nylon suture previously tied to the SF-IOL haptic plug was removed. A BSS/air exchange was performed, and fibrin glue (Tisseel Glue^®^, Baxter, Deerfield, IL, USA) was applied to close the conjunctiva above nasal and temporal scleral pockets.

#### 2.1.2. Follow-Up Examinations

At FU evaluations, a visual acuity examination and intra ocular pressure (IOP) and biomicroscopic examination were performed. All demographic and clinical parameters are summarized in Table 1. The AI-SF IOL complex remained well positioned throughout the follow-up period, with no intraocular or extraocular complications observed. The patient expressed satisfaction with both the visual and esthetic outcomes (Figure 1D,E).

### 2.2. CASE #2

A 34-year-old male patient presented with a mid-peripheral corneal perforation, extending from 11 to 12 o’clock, and iris prolapse, resulting from the thinning of a post-infectious central corneal leucoma in OD. Emergency penetrating keratoplasty was performed under general anesthesia. At the 5-month FU, biomicroscopy revealed a clear corneal flap, partial aniridia, phacodonesis, vitreous prolapse into the anterior chamber, and a flat, attached retina. Based on these findings, a decision was made to proceed with a vitrectomy and phacoemulsification, without IOL implantation, under peribulbar anesthesia. At 3 months FU, biomicroscopic examination revealed a clear corneal flap, partial aniridia, aphakia, and attached retina. It was subsequently decided to proceed with a combined implantation of an SF-IOL and a custom AI to allow full anatomical recovery.

#### 2.2.1. Surgical Procedure

**Figure 4:**
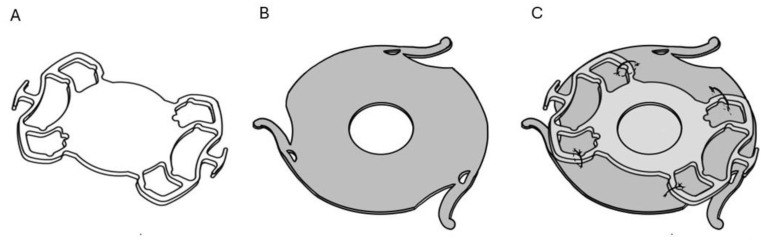
Case #2. Schematic representation of (**A**) SF-IOL, (**B**) artificial iris Reper, model C, and (**C**) the artificial iris secured to the four haptics of the scleral-fixated IOL using four sutures.

The assembly process of the AI-SF IOL complex was achieved as follows: the SF-IOL (+ 25 D, Fil SSF^®^, SOLEKO Spa, Rome, Italy, material: hydrophilic acrylic) was fixed to the posterior surface of the AI REPER^®^, model C, (Ophetec, Groningen, The Netherlands), using four 9/0 polypropylene sutures (PROLENE^®^, Ethicon LLC, San Lorenzo, PR, USA). Each suture ran within the iris implant material at the optic–haptic junction and around the four IOL haptics on both sides (Figure 4).

AI iridectomy and customization were not performed.

The surgical procedure mirrors that of case #1, but with several key differences. A 5.5 mm superior scleral incision was made with a 5.5 mm blade due to the presence of the transplanted corneal graft, aiming to minimize surgically induced astigmatism. The AI-SF IOL complex was folded in half and gently inserted through the superior incision into the anterior chamber using forceps. The SF-IOL haptics were then maneuvered into the scleral pocket at the 3 o’clock and 9 o’clock positions using the forceps.

#### 2.2.2. Follow-Up Examinations

**Figure 5:**
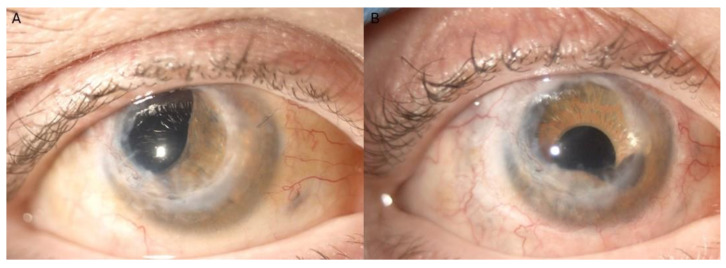
Anterior segment of case #2. (**A**) The corneal graft following perforating keratoplasty appears clear, with aniridia and aphakia. (**B**) Postoperative image obtained two weeks following AI-SF IOL complex implantation. At the 4 o’clock position, a blood clot undergoing resorption is visible.

During FU evaluations, visual acuity, IOP, and biomicroscopic examinations were performed. All demographic and clinical parameters are summarized in Table 1. The AI-SF IOL complex remained stable and well positioned throughout the FU period, with no intraocular or extraocular complications observed. The patient expressed satisfaction with both the visual and esthetic outcomes (Figure 5).

## 3. Discussion

The management of aphakic eyes with extensive iridodialysis or partial aniridia poses a significant surgical challenge. The primary objective is to restore visual acuity and improve the patient’s quality of life, while minimizing complications and the need for additional surgeries. The AI in cases of aniridia and aphakia has been implanted using various surgical techniques. AI has been positioned in the capsular bag, in conjunction with a capsular tension ring, or in the sulcus sutured to the sclera [9,10]. These procedures have also been carried out in pseudophakic eyes and concurrently with cataract extraction [11]. To our knowledge, these cases represent the first reported instances of a custom AI being sutured to an SF-IOL and secured as a single unit to the sclera.

### 3.1. Artificial Iris Trimming

Various studies have provided differing recommendations regarding the indications and optimal extent of trephination for the AI. For example, Bonnet et al. advocated for using larger AI diameters to avoid trephination whenever possible [12], whereas Rickman et al. recommended subtracting 0.5–1.0 mm from the WTW measurement [13]. In contrast, Nino Frisina et al. did not customize the AI based on the WTW measurement, as the Reper C AI model they utilized incorporates three haptics, eliminating the need for trimming [14].

In our experience, the decision to trim the AI differed between our two cases. In case #1, the AI was trimmed to a diameter of 11 mm, subtracting 0.8 mm from the measured WTW distance. In case #2, the AI was not trimmed, since we employed the Reper C AI model, whose three haptics inherently prevented the closure of the irido-corneal angle.

### 3.2. Corneo-Limbal vs. Corneo-Scleral Incision

The corneo-scleral incision used for secondary IOL implants is designed to minimize postoperative astigmatism. The incision size depends on the type of IOL and artificial iris implanted, ranging from 7.0 mm for the injection of a folded AI-SF IOL complex to 1.8 mm for cases where a fiber-free AI is implanted [7]. In case #1, a corneo-limbal incision was performed despite the potential for postoperative astigmatism, due to a previous superior scleral laceration. The Customflex^®^ AI with fibers was chosen over the fiberless model to enhance suture stability. However, because the AI with fibers is less flexible, an 8.0 mm incision was required to insert a partially folded AI-SF IOL complex, allowing it to slide into the AC [15]. In case #2, the AI-SF IOL complex was completely folded in half and inserted through a 5.5 mm incision. Due to the presence of a previously transplanted corneal flap, a corneoscleral incision was necessary.

### 3.3. IOL Models and Fixation Technique

Various techniques for implanting an IOL and AI in eyes without capsular support have been described in the literature. Yoeruek, E. et al. sutured the AI to a three-piece IOL, and the IOL was fixated to the sclera using a “Z” suture technique, in combination with PK surgery [16]. Mayer, C. et al. described six surgical techniques, two of which are particularly relevant to our procedure. The first is the “sandwich” technique, where the AI is sutured to a single-piece IOL. In this approach, the haptics are trimmed, and the AI is fixated to the sclera at the 3 and 9 o’clock positions using sutures. The second technique involves securing the AI to an SF-IOL. In this approach, the SF-IOL is sutured to the sclera at the 3 and 9 o’clock positions, while the AI is attached to the sclera at the 6 and 12 o’clock positions using sutures [7]. Additionally, another surgical technique has been proposed, the Canabrava Double-Flanged Technique, in which an artificial iris (REPER^®^ C model) with an integrated IOL is fixated to the sclera using its three haptics [8]. The Canabrava technique was also used to implant a combined AI and single-piece IOL complex [17]. Alternatively, Muth, D.R. et al. described the use of an AI sutured to a three-piece IOL implanted using the Yamane technique [18].

Additional techniques have been reported, including the fixation of a single-piece pinhole IOL to the sclera and the suturing of an AI to an iris-fixated IOL or directly to the sclera [19,20].

In our two cases, we opted for an SF-IOL due to its proven stability and lower risk of tilting compared to other implants [10,21]. Scleral fixation was performed without sutures by creating scleral pockets, thereby reducing the risk of conjunctival abrasion [22]. In both cases, the injection process was facilitated by the use of nylon loops, which prevented the complex dislocation into the vitreous chamber (VC) and minimized the risk of retinal damage.

### 3.4. Postoperative Complications

Postoperative complications associated with both SF-IOL and AI implants have been widely documented in the literature. Commonly reported complications include macular edema, hyphema, vitreous hemorrhage, chronic anterior inflammation, hypotony, hypertension, dislocation of the AI or IOL, IOL haptic exposure, endophthalmitis, retinal detachment, endothelial cell count loss, and Residual Iris Retraction Syndrome (RITS) [23,24,25]. In our two cases, no intraoperative or postoperative complications were observed. We attribute this outcome to the minimized intraocular maneuvers and the sutureless fixation of the AI-SF IOL, which likely helped prevent both anterior and posterior inflammation. The stability of the SF-IOL, achieved through scleral pocket fixation, further enhanced AI stability, reducing the risk of complications such as implant instability or IOL haptic exposure [21]. Additionally, the removal of residual iris tissue prevented the occurrence of RITS throughout the follow-up period, and no cases of endothelial corneal decompensation were observed.

Moreover, although iridectomies are frequently reported in the literature, they were not performed in our two cases [7]. In case #1, the AI was customized based on the WTW distance to ensure adequate aqueous humor flow. In case #2, the AI was left unmodified, as the three haptics of the Reper AI model C prevented the irido-corneal angle closure. Gonioscopic examination, in both cases, revealed an open angle with maintained communication between the VC and AC, and no acute or chronic increases in IOP was observed.

Despite these favorable outcomes, our cases have several limitations. The small sample size does allow for the standardization of the surgical technique. Additionally, the short follow-up period of up to 7 months does not rule out the possibility of long-term complications. Another limitation is the absence of pre- and postoperative endothelial cell counts, which would have been valuable in assessing endothelial damage resulting from AI-SF IOL complex implantation.

## 4. Conclusions

This modified AI-SF IOL technique successfully integrates artificial iris and intraocular lens implantation, offering both functional and esthetic restoration for aphakic eyes with extensive iridodialysis or aniridia. This procedure showed reproducibility across different AI models, while minimizing intraoperative complications. Further studies with larger cohorts and extended follow-up periods are encouraged to validate these findings and assess long-term outcomes across diverse patient populations.

## Figures and Tables

**Table 1:** Ophthalmic findings at baseline and after surgical procedures.

		Case #1	Case #2
Gender		Female	Male
Age		76	34
Post Emergency Surgery FU	IOP (mmHg)	12	10
	UCVA (LogMAR)	2.0	2.0
	BCVA (LogMAR)	1.0	1.0
	RC (Sf. Cyl/axis)	+12.0 −6.25/100°	−2.0 −3.0/0°
Post 2° Surgery FU	IOP (mmHg)	-	17
	UCVA (LogMAR)	-	1.8
	BCVA (LogMAR)	-	0.4
	RC (Sf. Cyl/axis)	-	+14.0 −2.0/5°
Post AI-SF IOL Implant Surgery, FU at 7 months	IOP (mmHg)	14	17
	UCVA (LogMAR)	0.8	1.0
	BCVA (LogMAR)	0.2	0.3
	RC (Sf. Cyl/axis)	+1.0 −3.50/75°	−2.0 −3.0/175°

Abbreviations: FU = follow-up, AI = artificial iris, SF IOL = scleral-fixated intra ocular lens, IOP = intraocular pressure, UCVA = uncorrected visual acuity, BCVA = best corrected visual acuity, RC = refractive correction.

## Data Availability

The data that support the findings of this study are available from the corresponding author upon reasonable request.
